# Case Report: Hyperbilirubinemia in Gilbert Syndrome Attenuates Covid-19-Induced Metabolic Disturbances

**DOI:** 10.3389/fcvm.2021.642181

**Published:** 2021-02-17

**Authors:** Hayder M. Al-kuraishy, Ali I. Al-Gareeb, Saleh M. Abdullah, Natália Cruz-Martins, Gaber El-Saber Batiha

**Affiliations:** ^1^Department of Clinical Pharmacology and Medicine, College of Medicine, Al-Mustansiriya University, Baghdad, Iraq; ^2^Department of Medical Laboratory Technology, Faculty of Applied Medical Sciences, Jazan University, Jazan, Saudi Arabia; ^3^Faculty of Medicine, University of Porto, Porto, Portugal; ^4^Department of Metabolism, Nutrition and Endocrinology, Institute for Research and Innovation in Health (i3S), University of Porto, Porto, Portugal; ^5^Laboratory of Neuropsychophysiology, Faculty of Psychology and Education Sciences, University of Porto, Porto, Portugal; ^6^Department of Pharmacology and Therapeutics, Faculty of Veterinary Medicine, Damanhour University, Damanhour, Egypt

**Keywords:** gilbert syndrome, SARS-CoV-2, hyperbilirubinemia, COVID-19, metabolic disease

## Abstract

Gilbert syndrome (GS) is a liver disorder characterized by non-hemolytic unconjugated hyperbilirubinemia. On the other hand, Coronavirus disease 2019 (Covid-19) is a recent viral infectious disease presented as clusters of pneumonia, triggered by the severe acute respiratory syndrome-coronavirus 2 (SARS-CoV-2). Little is known on the association between SARS-CoV-2 and GS, despite different studies have recently stated a link between hyperbilirubinemia and SARS-CoV-2 severity. In this case-report study we described a 47-year-old man, a known case of GS since the age of 4, presented to the emergency department with fever (39.8°C), dry cough, dyspnea, headache, myalgia, sweating and jaundice diagnosed with Covid-19-induced pneumonia. Interestingly, GS patient exhibited a rapid clinical recovery and short hospital stay compared to other SARS-CoV-2 positive patient, seeming that hyperbilirubinemia may exert a protective effect of against Covid-19 induced-cardiometabolic disturbances. Data obtained here underlines that the higher resistance against Covid-19 evidenced by the GS patient seems to be due to the antioxidant, anti-inflammatory, and antiviral effects of unconjugated bilirubin.

## Introduction

Gilbert syndrome (GS) is a chronic liver disorder characterized by non-hemolytic unconjugated hyperbilirubinemia due to defect in the hepatic uptake of unconjugated bilirubin, which was first described by Augustin Gilbert in 1901 (1). GS is also called simple familial jaundice or icterus intermittent juvenilis, affects 5–10% of general population, being most common in male (2). Clinically, GS is presented with mild recurrent jaundice, fatigue and abdominal pain provoked by stress, infection, and menstruation. GS results from reduction in bilirubin uridine diphosphate glucuronyltransferase enzyme activity due to mutation in the UGT1A1 gene. There are more than 100 variants of UGT1A1 gene associated with GS phenotype, and generally, there is no effective treatment for GS, despite phenobarbital may be used in severe cases (3). Previously, Maruhashi et al. (4) reported that hyperbilirubinemia in GS is associated with a cardioprotective effect attributed to the antioxidant and vasodilator effects of bilirubin.

On the other hand, coronavirus disease 2019 (Covid-19), a recent viral infectious disease presented as clusters of pneumonia and caused by the severe acute respiratory syndrome coronavirus-2 (SARS-CoV-2), has triggered a huge attention among both medical and scientific communities with the intent of discovering an effective therapeutic agent (5). The clinical spectrum of Covid-19 is asymptomatic or mild flu-like illness in around 85%, mainly in young adults; however, 10% of cases develop a severe disease with risk of development of acute respiratory distress syndrome (ARDS) (6). However, in severe cases, Covid-19 may leads to extra-pulmonary manifestations, like acute cardiac injury, arrhythmias, acute kidney injury, acute brain injury, endocrine failure, multiple organ failure, metabolic disturbances, and even death (7). In this sense, as Covid-19 pandemic has full-grown public health issues, here we present a case-report study of a patient with GS who gets infected by the SARS-CoV-2. This case is particularly relevant regarding the ameliorative role of hyperbilirubinemia in GS patients during Covid-19 pneumonia.

## Case Report

### Presenting Concerns

A 47-year-old man, a known case of GS since age of 4-year, presented to the emergency department with fever, dry cough, dyspnea, headache, myalgia, sweating, jaundice, and generalized poor health condition without response to the empiric antibiotics and analgesics for about 3 days. Besides, a 53-years-old man presented with fever (38.9°C), cough, headache, malaise and sweating diagnosed as Covid-19 pneumonia was regarded as a control. Informed verbal consent was attained from both patients, and this study was approved (MRT 7 August 2020) by the Scientific Editorial Board in College of Medicine, Al-Mustansiriyia University, Baghdad, Iraq.

### Clinical and Laboratory Findings

**Figure 1:**
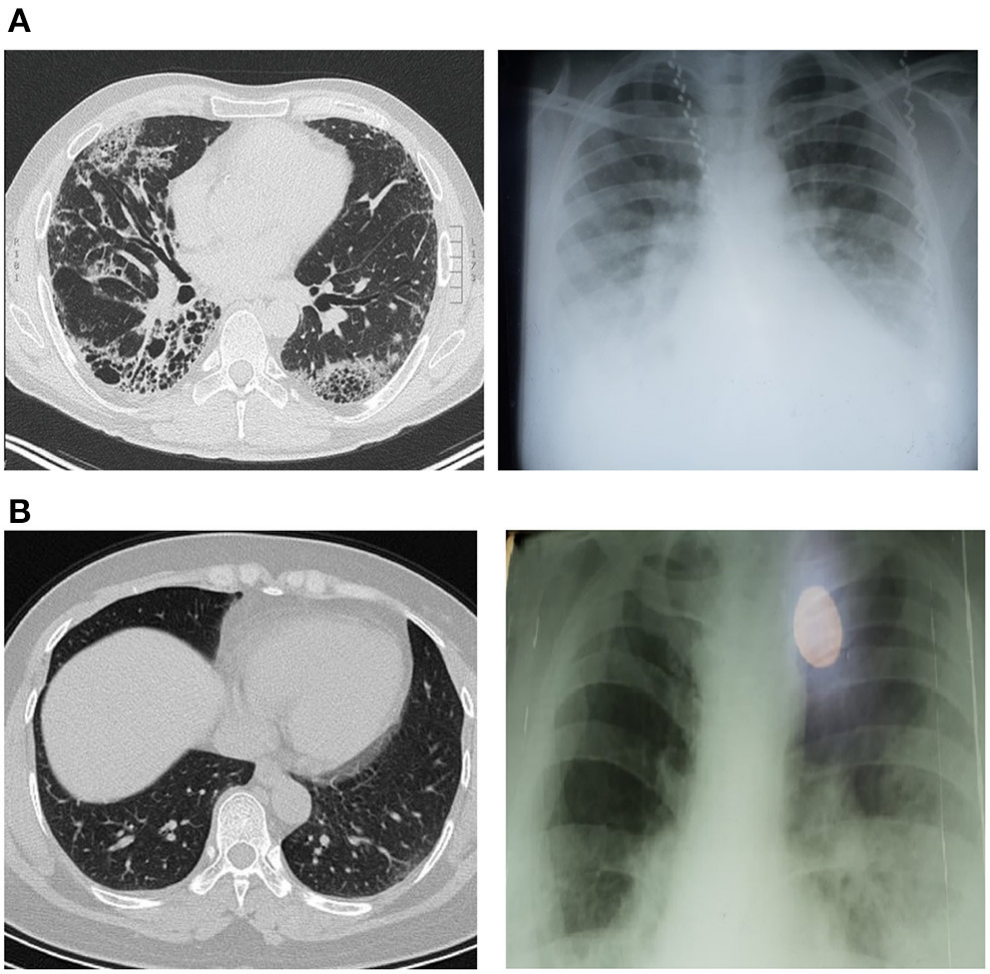
Chest X-ray and CT scan imaging showed bilateral ground-glass appearance; **(A)** Covid-19 patients only, **(B)** Covid-19 patients with Gilbert syndrome.

General physical examination showed a conscious and febrile patient (39.8°C), with jaundice and poor health status. His blood pressure was 140/90 mmHg, heart rate was 110 beats/min and body mass index (BMI) of 33.73 kg/m^2^ and hypoxemia (SaO_2_ 91%). Chest X-ray and chest computed tomography (CT) scan illustrated bilateral prominent bronchovascular marking and ground-glass opacities, respectively, suggestive of Covid-19-induced pneumonia (Figure 1). Radiological score was used to determine the radiological severity according to Wasilewski et al. (8).

Anti-SARS-CoV-2 antibody (IgM) was positive (2.9 U/mL) for Covid-19 patient with GS compared with (2.89 U/mL) for Covid-19 patient only, suggesting an acute SARS-CoV-2 infection in both. Complete blood count (CBC), fasting blood glucose (FBG), glycated hemoglobin (HbA1c), blood urea, serum creatinine, C-reactive protein (CRP), D-dimer, serum lactate dehydrogenase (LDH), and serum ferritin were done at the laboratory unit. Preliminary investigations showed high FBG (165 mg/dL), HbA1c (5.5%), total serum bilirubin (6.8 mg/dL), unconjugated bilirubin (6 mg/dL), conjugated bilirubin (0.8 mg mg/dL), and high white cell counts (16.073/μL) with lymphopenia (9.12 μ/L). Similarly, the inflammatory biomarkers were increased in regard to reference ranges. D-dimer (14.000 ng/mL), CRP (243 mg/L), ferritin (654 ng/mL), and LDH (674U/L).

Liver function test and ultrasonography imaging were done to exclude liver injury. Taken together, clinical findings, radiological examinations and laboratory findings of this GS patient with Covid-19 were compared with a matched COVID-19 patient without GS at time of hospitalization (Table 1).

**Table 1:** Cardiometabolic and inflammatory profiles of GS patient COVID-19 positive compared to a control patient at time of admission.

**Variables**	**Reference range**	**COVID-19 patient with GS**	**COVID-19 patient**	**% Difference**
BMI (kg/m^2^)	20–25	33.73	34.71	2.86
SBP (mmHg)	110–120	140	153	8.87
DBP (mmHg)	70–90	90	92	2.19
Covid-19 IgM (U/mL)	0.9–1.1	2.9	2.89	0.34
Covid-19 IgG (U/mL)	0.9–1.1	0.00	0.00	0.00
SaO_2_%	95–99	91	89	1.11
TSB (mg/dL)	0.2–1.0	6.8	0.8	157.89
Conjugated bilirubin (mg/dL)	0.1–0.3	0.8	0.7	13.33
Un-conjugated bilirubin (mg/dL)	0.1–0.7	6.0	0.1	193.44
FBG (mg/dL)	70–90	165	199	18.68
HbA1c (%)	4.5–5.5	5.5	5.9	7.01
Blood urea	20–40	41	39.7	3.22
Serum creatinine	0.5–1.5	1.2	1.1	8.69
CRP (mg/L)	0.5–200	243	422	53.83
D-dimer (ng/mL)	50–10.000	14.000	22.000	44.44
Ferritin (ng/mL)	20–250	654	907.84	32.50
LDH (U/L)	230–460	674	795.21	16.50
Hb (g/dL)	12–14	13.8	14.36	3.97
WBC (μ/L)	4,000–11,000	16.073	15.74	2.09
Lymphocytes %	20–40	9.12	7.53	19.09
Neutophils %	40–80	85.31	89.45	4.73
Radiological score	1–5	4	5	22.22

*Data presented as number and %, BMI, body mass index; SBP, systolic blood pressure; DBP, diastolic blood pressure; TSB, total serum bilirubin; FBG, fasting blood glucose; HbA1c, glycated hemoglobin*.

Both patients were treated with the analgesic acetaminophen (500 mg/day), azithromycin (500 mg/day) for the first 5 days, ivermectin (12 mg/day), famotidine (40 mg/day), soluble insulin (10 units) 3 times/day, and montelukast (10 mg/day). Besides oxygen therapy by high flow nasal cannula for 10 days, patients also received subcutaneous enoxaparin (60 mg/day) during the hospitalization period as a prophylaxis against venous thromboembolism.

### Follow-Up and Outcomes

Following 3 weeks of management, all laboratory investigations, radiological, and clinical findings return to normal except of unconjugated bilirubin (Table 2) and the patient was discharged to home. Particularly, the GS patient showed a rapid clinical improvement as compared to the Covid-19 patient without GS during the hospitalization period.

**Table 2:** Cardiometabolic and inflammatory profiles of GS patient COVID-19 positive compared to a control patient at time of discharge.

**Variables**	**Reference range**	**Covid-19 with GS**	**Covid-19 patient**	**% Difference**
BMI (kg/m^2^)	20–25	32.65	34.71	6.11
SBP (mmHg)	110–120	119	143	18.32
DBP (mmHg)	70–90	79	82	3.72
Covid-19 IgM (U/mL)	0.9–1.1	0.9	0.89	1.11
Covid-19 IgG (U/mL)	0.9–1.1	7.84	6.01	26.42
SaO_2_%	95–99	98	95	3.10
TSB (mg/dL)	0.2–1.0	3.4	0.8	123.81
Conjugated bilirubin (mg/dL)	0.1–0.3	0.4	0.7	54.54
Un-conjugated bilirubin (mg/dL)	0.1–0.7	3.0	0.1	187.09
FBG (mg/dL)	70–90	95	179	61.31
HbA1c (%)	4.5–5.5	5.5	5.9	7.01
Blood urea	20–40	33	34.7	5.02
Serum creatinine	0.5–1.5	1.3	1.2	8
CRP (mg/L)	0.5–200	22	122	138.88
D-dimer (ng/mL)	50–10.000	452	631.71	33.16
Ferritin (ng/mL)	20–250	105	207.84	65.74
LDH (U/L)	230–460	321	395.21	20.72
Hb (g/dL)	12–14	13.8	14.36	3.97
WBC (μ/L)	4,000–11,000	8.832	9.44	6.65
Lymphocytes %	20–40	33.7	22.53	39.72
Neutophils %	40–80	72.88	80.45	9.87
Radiological score	1–5	1	2	66.66

*Data presented as number and %, BMI, body mass index; SBP, systolic blood pressure; DBP, diastolic blood pressure; TSB, total serum bilirubin; FBG, fasting blood glucose; HbA1c, glycated hemoglobin*.

An outpatient follow-up through mobile dial-up within 2 weeks following discharge disclosed a complete recovery and the GS patient returned to his prior physical fitness and normal daily activities.

## Clinical Course Summary

At time of hospitalization, both Covid-19 patients with or without GS presented comparable clinical presentations, like fever, headache, sweating, dry cough, fatigue, and generalized poor health status. However, these clinical features were less severe in Covid-19 patient with GS compared with Covid-19 patient only. In addition to high serum levels of unconjugated bilirubin in Covid-19 patient with GS, both laboratory and radiological findings were better as compared with Covid-19 patient only. In the management period, patients received the same course of supportive therapy, antibiotics, anticoagulants, and other drugs. During hospitalization period, the fasting blood glucose (FBG) was elevated in both Covid-19 patients (FBG = 165 mg/dL in GS, 199 mg/dL in Covid-19 control), managed through using soluble insulin subcutaneously 10 IU/day with frequent monitoring of FBG. In particular, Covid-19 patient with GS presented with a less needed for oxygen therapy compared with control Covid-19 patients who was more dependent on oxygen therapy. Near the end of hospitalization period, Covid-19 patient with GS showed a rapid clinical improvement as compared to the Covid-19 patient without GS. At the third week of disease management, clinical, radiological and laboratory findings were re-evaluated. All investigations and clinical findings return to normal with exception of unconjugated bilirubin, which remained higher in Covid-19 patient with GS (3 mg/dL) as compared with that in control Covid-19 patient (1 mg/dL). Both patients were discharged to home with complete recovery and returned to normal daily activities.

## Discussion

To our knowledge, this is the first reported case study of Covid-19 in a patient with GS. The GS patient with Covid-19 showed a rapid clinical improvement and short hospital stay as compared with a Covid-19 patient. Indeed, it has been proven that bilirubin exerts potent antioxidant effects which might alleviates Covid-19 induced-oxidative stress (9). Also, it has been reported that bilirubin has cardioprotective effects, improves endothelial function and provokes the nitric oxide (NO) release (10), thus, preventing from endothelial dysfunction and cardiovascular complications in COVID-19 (11), as evident of hypertension in Covid-19 case compared to GS patient with Covid-19. Unfortunately, oxidative stress profile and endogenous antioxidant capacity were not measured in the present study to confirm the antioxidant potential of unconjugated bilirubin in Covid-19.

Liu et al. (12) found that SARS-CoV-2 infection leads to down-regulation of angiotensin converting enzyme 2 (ACE2) causing a reduction in the vasodilator angiotensins (Ang 1–7 and Ang 1–9) and augmenting of vasoconstrictor angiotensin II (AngII). These changes *per se* lead to acute lung injury (ALI), cardiovascular and metabolic disturbances in Covid-19 patients. Recently, Novák et al. (13) reported that high bilirubin levels attenuate the metabolic disorders through inhibition and attenuation of renin-angiotensin system (RAS). Besides, bilirubin has a protective effect against experimental ALI through inhibition of ischemic-reperfusion injury and exerting anti-proliferative effects (14). Therefore, high serum bilirubin level in patients with GS may lessen ALI and the development of ARDS through attenuation of AngII induced-pulmonary vasoconstriction and hyper-inflammation (15). These findings might explain a lower CT score 4 in Covid-19 with GS as compared with control Covid-19 score 5.

Lin et al. (16) also illustrated that bilirubin inhibits the nod-like receptor pyrin3 (NLRP3) inflammasomes over-activation through myeloperoxidase inhibition and subsequent reduction of inflammatory cytokines release. Thereby, high serum bilirubin levels in patients with GS may attenuate the development of cytokine storm during Covid-19 progression via inhibiting the release of interleukin (IL)-6, tumor necrosis factor (TNF)-α and IL-1β (17). These findings might explain the low rate of inflammatory biomarkers in GS patient with Covid-19 compared to the Covid-19 patient without GS. These protective effects of high bilirubin in GS are lacking in patients with Covid-19 pneumonia without GS. It has been shown that uncontrolled high pro-inflammatory cytokines, oxidative stress and unrestrained activation of NLRP3 inflammasomes contribute for development of ALI and progression of Covid-19 severity (18, 19).

Interestingly, fasting blood glucose (FBG) was increased at time of admission due to SARS-CoV-2 induced insulin resistance and transient pancreatic β-cells dysfunction. (20). However, FBG seem to be lower in Covid-19 patient with GS, since high unconjugated bilirubin in GS improves FBG and hyperinsulinemia through activation of peroxisome proliferative activated receptor alpha (PPAR-α) (21).

On the other hand, Santangelo et al. (22) disclosed that endogenous bilirubin has antiviral property against human herpes simplex virus type 1 (HSV-1), hepatitis C virus and enterovirus EV71 via up-regulation of mitogen activated protein kinase (MAPK) and c-Jun N-terminal (JNK). Both of MAPK and JNK are involved in the replication and pathogenesis of SARS-CoV-2 and other coronaviruses (23). Therefore, bilirubin may be the future endogenous agent against SARS-CoV-2. Nonetheless, Liu et al. (24) found that serum bilirubin levels are correlated with Covid-19 induced-liver injury and hemolysis, but the author ignored the antioxidant and anti-inflammatory properties of bilirubin.

The present case-report study had some limitations, including genetic sequence genotype and genetic information of family of patient with GS was not evaluated, relevant past interventions were not recorded, and antioxidant profile was not estimated. Even though this study is regarded as a baseline for future clinical trials and large-scale prospective to confirm the protective effect of unconjugated bilirubin against Covid-19.

## Conclusion

Taken together, data obtained in this case report study shed light on the new modality for COVID-19 therapy through modulation of bilirubin metabolism. As well, high bilirubin levels in the GS patient with COVID-19 conferred a protective effect against COVID-19-derived cardiometabolic disturbances. In fact, the GS patient revealed higher resistance against COVID-19 associated cardiometabolic disturbances compared to the other COVID-19 patient without GS, directly linked to the antioxidant, anti-inflammatory and antiviral effects of unconjugated bilirubin. However, we cannot sketch any definitive conclusion from our observation; thus prospective, randomized, controlled studies are recommended in this regard.

## Data Availability Statement

The raw data supporting the conclusions of this article will be made available by the authors, without undue reservation.

## Ethics Statement

The studies involving human participants were reviewed and approved by Al-Mustansiriyia University. The patients/participants provided their written informed consent to participate in this study. Written informed consent was obtained from the individual(s) for the publication of any potentially identifiable images or data included in this article.

## Author Contributions

All authors listed have made a substantial, direct and intellectual contribution to the work, and approved it for publication.

## Conflict of Interest

The authors declare that the research was conducted in the absence of any commercial or financial relationships that could be construed as a potential conflict of interest.
